# Knowledge domain and research trends for Gestational Diabetes Mellitus and nutrition from 2011 to 2021: a bibliometric analysis

**DOI:** 10.3389/fnut.2023.1142858

**Published:** 2023-07-05

**Authors:** Zhefang Hu, Qianyi Chen, Man Luo, Yanwei Ren, Jianyun Xu, Lijun Feng

**Affiliations:** ^1^Department of Clinical Nutrition, Sir Run Run Shaw Hospital, Medical School of Zhejiang University, Hangzhou, Zhejiang, China; ^2^Department of Obstetrics, Sir Run Run Shaw Hospital, Medical School of Zhejiang University, Hangzhou, Zhejiang, China; ^3^School of Art and Design, Taizhou University, Taizhou, Zhejiang, China

**Keywords:** gestational diabetes mellitus, dietary, nutrient, bibliometric analysis, diagnostic criteria

## Abstract

**Objective:**

Nutrient management and lifestyle changes are the frontlines of treatment for all pregnant women diagnosed with Gestational Diabetes Mellitus (GDM). This study aimed to identify the global research architecture, trends, and hotpots of GDM and nutrition.

**Methods:**

We obtained publications from the sub-databases of Science Citation Index Expanded and Social Science Citation Index sourced from the Web of Science Core Collection database on January 4, 2022, using publication years between 2011 and 2021. CiteSpace software, VOSviewer, and Microsoft Excel 2019 were used to conduct the bibliometric analyses.

**Results:**

A growing publication trend was observed for GDM and nutrition, and this field has great potential. More GDM and nutrition research has been conducted in developed countries than developing countries. The top three authors with a high publication frequency, co-citations, and a good h-index were from the United States. There were the four studies of randomized controlled trials (RCTs) or meta-analyses of RCTs, as well as one review in the top five items of cited literature. Keywords were categorized into four clusters based on the keywords visualization.

**Conclusion:**

It is important to strengthen the collaboration between nations of different economies to produce more high-quality research on GDM and nutrition. It may be beneficial to further study the etiology, diagnosis, and treatment of GDM based on current results to provide a new perspective on GDM and nutrition.

## 1. Introduction

Gestational Diabetes Mellitus (GDM) describes a disorder of glycometabolism that develops during pregnancy but does not meet the diagnostic criteria for diabetes mellitus (1), affecting up to 18% of all birth-giving women (2). During the last decade, there was a growing concern about GDM in the public health area (3, 4), and associated with obesity and overweight, the prevalence of GDM is rapidly rising (5–7).

Nutrient management and lifestyle changes are the frontlines of treatment for all pregnant women diagnosed with GDM, regardless of the severity of their phenotypic profile (8–11). A proper nutrition program could obtain high-quality nutrients, gain adequate gestational weight, and grow healthy babies (12). It is important to note that approximately 70% of women can control GDM solely through diet and lifestyle changes (8). However, the best dietary strategies for GDM remain controversial (13), and the overall evidence quality of clinical trials involving dietary or nutrition therapy for GDM has been evaluated as low, or the bias risks are unclear in general (14, 15). In addition, the debate still revolves around the diagnostic criteria for GDM and whether it is clinically effective in treating women with diagnosed GDM (13, 16–18). As outlined above, it is necessary to acquire detailed knowledge of the global research architecture, trends, and hotpots of GDM and nutrition.

Bibliometric analyses are statistical methods for quantitative analyses based on the public literature database. They can be used to demonstrate the evolution process of a certain knowledge field by drawing a knowledge map and predicting the future frontiers (19–21). Although research on GDM and nutrition is extensively available globally, as far as we know, limited studies use a bibliometric analysis to evaluate all aspects of our study topic. Therefore, in this study, we performed the bibliometrics of the publications pertaining to GDM and nutrition published between 2011 and 2021. Using our results, we aim to present an overview of the achievements, hot spots, and potential future directions in this field of research to provide support for fellow scholars looking to stay up-to-date with the latest developments in this area.

## 2. Methods

### 2.1. Data sources and search strategy

As a data source, we preferred the Web of Science (WoS) database to the PubMed database because the publications listed in the WoS are accompanied by a wide range of bibliographic data readily accessible (22, 23). Additionally, the Web of Science Core Collection (WoSCC) database, the high-quality literature database of the WoS, can provide the most influential and reliable information in light of continuous and dynamic updates (22, 23). We obtained the publications from the sub-databases of the Science Citation Index Expanded (SCI-E) and Social Science Citation Index (SSCI) sourced from the WoSCC database on January 4, 2022. All searches and data collections were completed within the same day. The search strategy was “TS = (gestational diabetes OR GDM OR gestational diabetes mellitus) AND TS = (dietary OR nutri*).” According to the inclusion criteria, 1,727 publications were finally included in our study.

### 2.2. Inclusion criteria

The publication language was “English,” and only “articles” and “reviews” were selected. The analyzed timeframe for research covered the years between January 1, 2011, and December 31, 2021.

### 2.3. Data statistics and indicators

We used CiteSpace (V. 6.2.R3) software, invented by Professor Chaomei Chen (24) (School of Information Science and Technology, Drexel University, Philadelphia, PA, United States), to perform the Bibliometric Visual Analysis. Several bibliometric studies can be conducted using this software, such as a collaboration network analysis, an author co-citation analysis, and document co-citations (25–27). VOSviewer (version 1.6.6; Leiden University Center for Science and Technology Studies, Leiden, Netherlands) was also used to visualize the bibliometric networks, such as through a keyword analysis (28). Microsoft Excel 2019 software was used to analyze the trend of publications in each year.

The analysis indicators included publication number, average citation per publication, countries, institutions, journals, authors, the Hirsch Index (H-index, defined as the number of papers with citation number ≥ h) (29), and the Impact Factor (IF) within 5 years average. In the network graph, different nodes represent various elements, such as institutions, countries, and authors. The circles indicate the frequency or number of publications; the bigger the circles, the more frequent the publications (24). The centrality indicates a node’s role in the knowledge network and its influence on other nodes (24). A great centrality index increases the likelihood that key nodes will arise in the network (24).

## 3. Results

### 3.1. The annual publication distribution map, citation frequency, and trends

**Figure 1:**
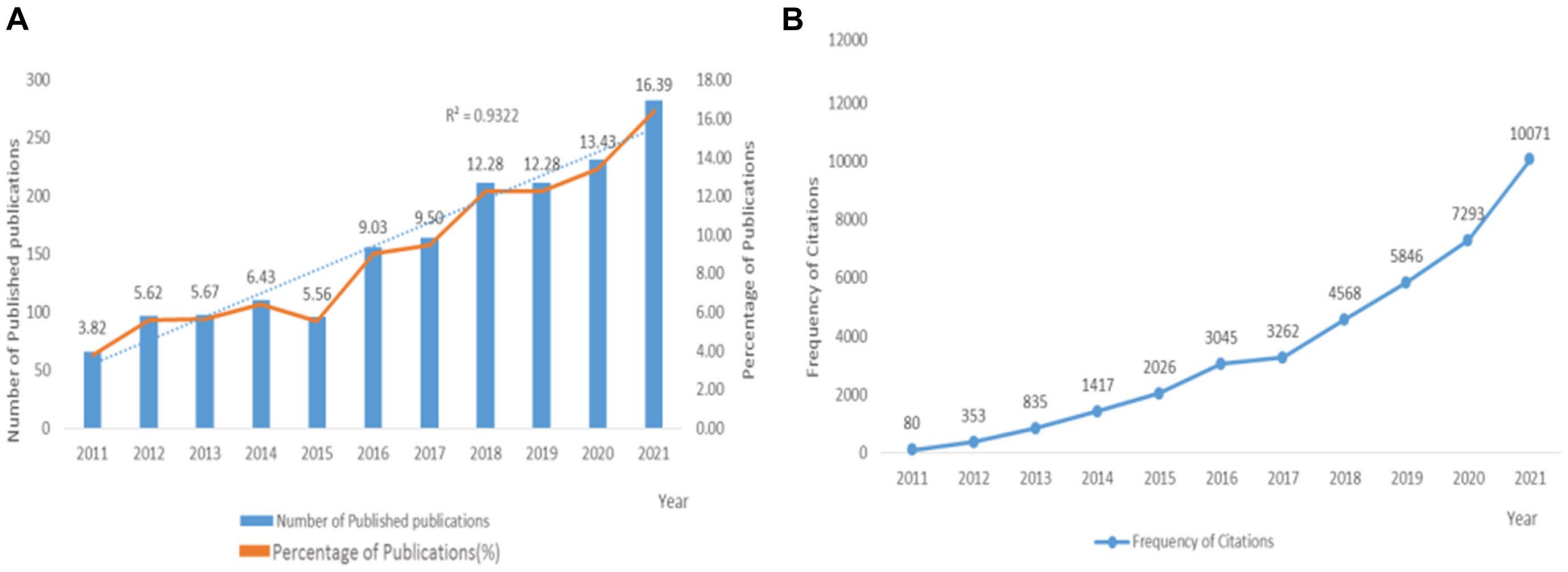
Global number of publications and frequency of citations in the field of GDM and nutrition from 2011 to 2021. **(A)** The annual number of the published publications and their percentage; **(B)** the annual citation frequencies of publications.

In total, 1,727 papers met the inclusion criteria, which includes 1,300 (75.27%) full-length articles and 427 (24.73%) review articles. A growing publication trend was observed from 66 in 2011 to 283 in 2021. In 2015, the number of publications decreased (96 papers). Since 2016, the number of publications has increased steadily, and the percentage of publications was the highest in the most recent 3 years (42.1%). In this study, the association between the publication year and the number of publications was described using Goodness-of-fit Tests (30). There was a significant correlation between the number of studies and the year with a high coefficient of determination (*R*^2^ = 0.932), and the details are illustrated in Figure 1A.

By the end of the search date, these publications had been mainly cited 40,160 times, and the average number of citations for publications was 23.25. The annual citations on GDM and nutrition revealed a significant growth in two distinct periods, namely between 2015–2016 and 2020–2021, as illustrated in Figure 1B.

### 3.2. Distribution of country/region

**Figure 2:**
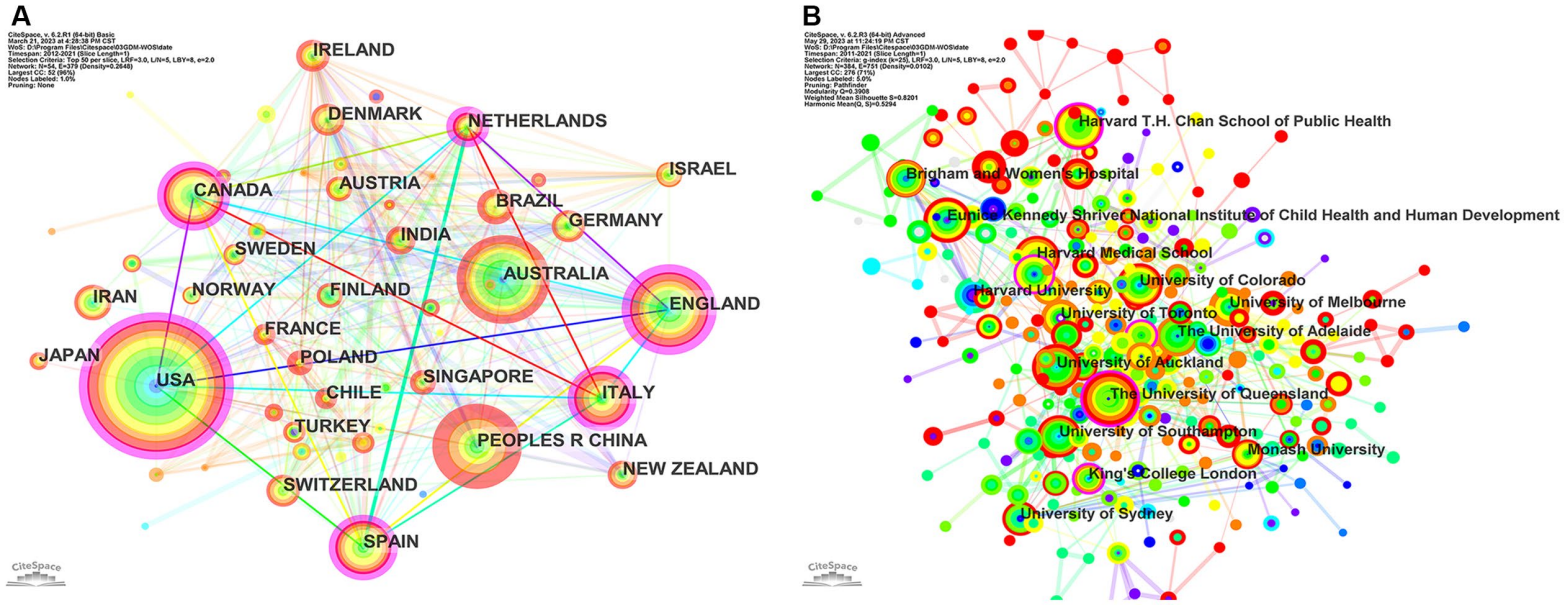
**(A)** Network map of GDM and nutrition co-countries/regions in the WoSCC database. There are 53 nodes, which means 53 countries/regions included, 377 nodes connections, and the network density is 0.2736. **(B)** Network map of GDM and nutrition co-institutions in the WoSCC database. There are 341 nodes, which means 341 institutions included, 1,268 nodes connections, and the network density is 0.0219. The rose-red outer circles indicate the centrality (≥0.1) of research cooperation in the countries/regions or institutions; the thicker the outer circles are, the closer the relationships of research cooperation among the countries/regions or institutions are established. Lines indicate the relationship of cooperation or co-occurrence among countries/regions or institutions.

The publications were distributed among 53 countries/regions when setting “Node Type” to “Country,” as illustrated in the network map of countries/regions (Figure 2). The highest frequency of published publications was in the United States (468), followed by China (222), Australia (220), and England (175). Betweenness centrality is one of the core concepts in CiteSpace, which means the level of closeness of research collaboration among counties or regions, and usually, no less than 0.1 represents the satisfactory cut-off value (25, 27). The high betweenness centrality countries were England (0.26), the United States (0.25), Australia (0.15), Spain (0.12), and Italy (0.11).

Overall, countries with a higher frequency of publications also have higher betweenness centrality. The United States was much better than other countries, whether for the frequency of published publications or the betweenness centrality in the field of GDM and nutrition. Notably, the frequency of publications in China was ranked second in the world in this field, but the betweenness centrality was as low as 0.04, demonstrating that China has not yet established good research cooperation relationships with other countries (Figure 2A; Table 1).

**Table 1:** The top 10 frequency of publications countries and institutions with the highest number of publications with centrality in the field of GDM and nutrition in the WoSCC database.

Country	Frequency of publications	Centrality	Institution	Frequency of publications
United States	468	0.25	University of Queensland	58
Australia	241	0.15	University of Auckland	44
People’s Republic of China	222	0.05	Eunice Kennedy Shriver National Institute of Child Health and Human Development	41
England	175	0.26	University of Southampton	38
Canada	133	0.1	University of Colorado	38
Spain	98	0.12	Harvard Med School	36
Italy	89	0.11	University of Adelaide	36
Denmark	60	0.05	Harvard T. H. Chan School of Public Health	32
Brazil	57	0.04	Brigham and Women’s Hospital	32
Netherlands	55	0.12	Harvard University	29

**Figure 3:**
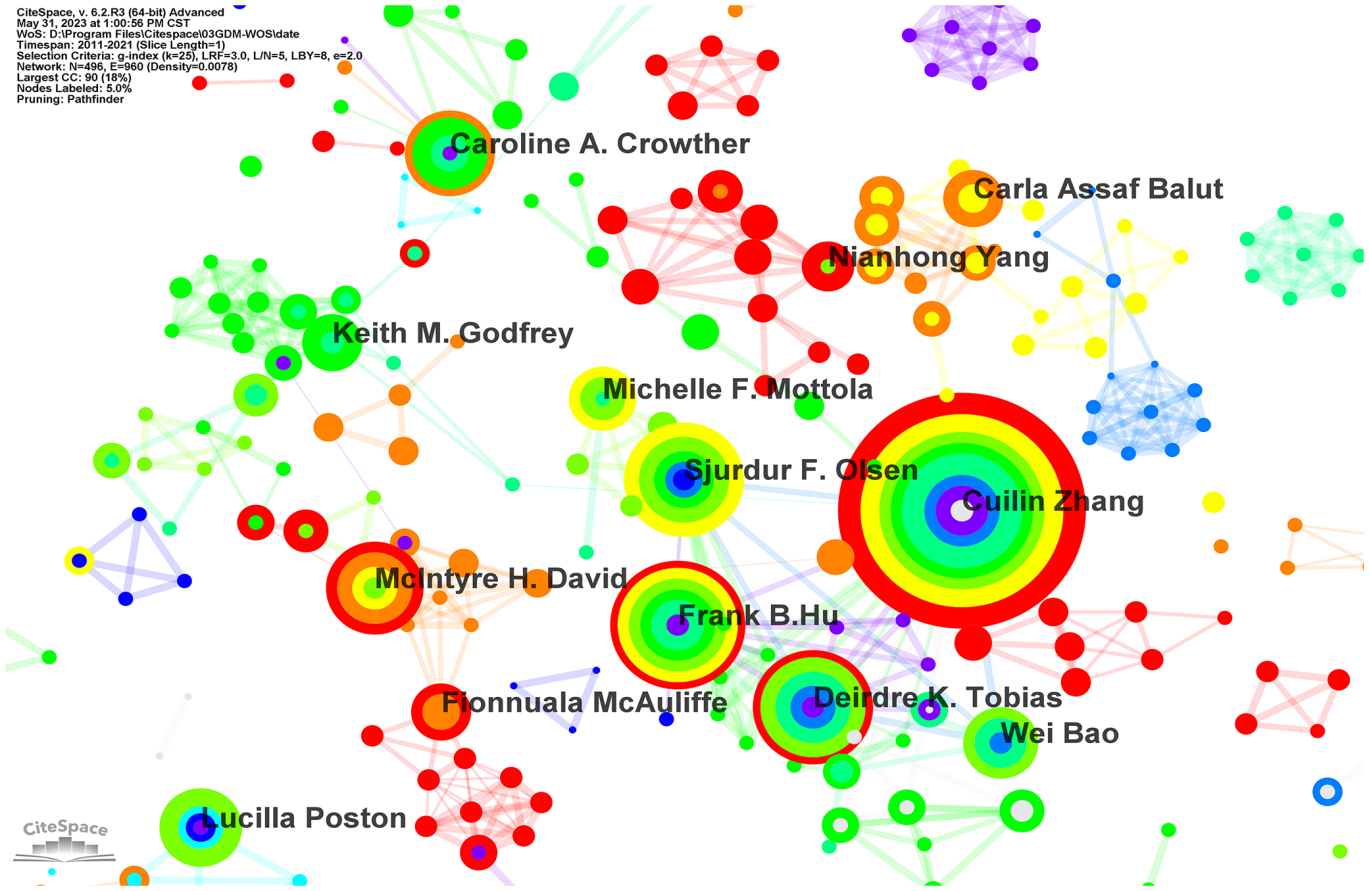
Network map of GDM and nutrition co-authorship in the WoSCC database. There are 417 nodes, which means 417 authors included, 989 nodes connections, and the network density is 0.0114. Absence of the authors whose betweenness centrality≥0.1.

There were 341 institutions that had published publications in the field of GDM and nutrition when setting “Node Type” to “Institution,” as illustrated in the network map of the institution (Figure 3). The highest frequency of published publications was the University of Queensland (31) in Australia, followed by the University of Auckland in New Zealand (32), and Eunice Kennedy Shriver National Institute of Child Health and Human Development in the United States (33) (Figure 2B; Table 1).

### 3.3. High-influence authors and author collaborations

In total, 417 authors participated in publications in the field of GDM and nutrition, as illustrated in the visualization map (Figure 3), with an average of 4.14 authors per publication. The CiteSpace statistics demonstrate that Cuilin Zhang was the most productive scholar with 33 publications, followed by Frank B Hu (23) and Lucilla Poston (21). The authors with the highest total citations were Cuilin Zhang (1721), Lucilla Poston (1688), and Deirdre K. Tobias (971). The highest average citations per paper were for Lucilla Poston (80.38), Deirdre K. Tobias (51.11), and Cuilin Zhang (49.17). Cuilin Zhang (20), Frank B. Hu (15), Lucilla Poston (14), and Deirdre K. Tobias (14) ranked as the first three highest H-index authors (see Table 2). Generally, the degree of cooperation was not close among the authors, the network density was 0.006, and close cooperation mainly existed among Cuilin Zhang, Frank B. Hu, Sjurdur F. Olsen, and Wei Bao, who produced more publications than the other authors.

**Table 2:** Top 10 influential authors in the field of GDM and nutrition in the WoSCC database.

Authors	Country	Frequency	Total citation	Average citation per paper	*H*-index
Cuilin Zhang	United States	35	1721	49.17	20
Frank B Hu	United States	23	1,015	44.13	15
Lucilla Poston	United States	21	1,688	80.38	14
Sjurdur F. Olsen	Denmark	20	623	31.15	13
Deirdre K. Tobias	United States	19	971	51.11	14
Caroline A. Crowther	Australia	19	929	48.89	13
Wei Bao	United States	19	534	28.11	13
H. David McIntyre	Australia	19	633	33.32	10
Michelle F. Mottola	Ireland	17	358	21.06	9
Keith M. Godfrey	England	16	671	41.94	11

### 3.4. Highly cited literature on nutritional interventions for the prevention or treatment of GDM

The citation frequency of a paper is a critical indicator of its status as a high-impact paper with substantial research results. The most frequently cited publications (25, 27), also known as “highly cited literature,” are the main focus of scholars’ attention. Papers with more than 50 citations were mainly concentrated from 2010 to 2016. Table 3 summarizes the titles, research methods, first author, the publishing year and journal, the main results and conclusions, the cited frequency, and the IF of the top five items of highly cited literature on nutritional interventions for the prevention or treatment of GDM. This table illustrates the four studies of RCT or meta-analyses of RCTs, as well as one review in the top five items of cited literature. Thangaratinam et al.’s (34) work earned the most citations (549). The topics explored include a variety of interventions based on nutrition and diet, probiotic supplements, vitamin D and a low-glycemic index (GI) diet. Notably, three reports were published in journals with an IF≥10 (*BMJ, Lancet Diabetes & Endocrinology, The Cochrane Database of Systematic Reviews*).

**Table 3:** The top five highly cited items of literature on nutritional interventions for the prevention or treatment of GDM in the WoSCC database.

Title	Research method	First author	Year	Journal	Result and conclusions	Citation	IF (average 5 years)
Effects of interventions in pregnancy on maternal weight and obstetric outcomes: meta-analysis of randomized evidence (34)	meta-analyses of RCTs	Thangaratinam S	2012	BMJ	Forty-four relevant randomized controlled trials (7,278 women) evaluating diet, physical activity, and a mixed approach. Dietary and lifestyle interventions resulted in the largest reduction in maternal GWG, with improved pregnancy outcomes compared with other interventions. Among the interventions, those based on diet are the most effective.	549	38.20
Effect of a behavioral intervention in obese pregnant women (the UPBEAT study): A multicenter, randomized controlled trial (32)	RCT	Poston L	2015	Lancet Diabetes and Endocrinology	The results suggest that a complex intervention addressing diet and physical activity in pregnant women with obesity is effective in reducing GWG. However, the complex intervention in women with obesity during pregnancy is not adequate to prevent gestational diabetes or to reduce the number of large-for-gestational-age infants.	420	29.79
Impact of maternal probiotic-supplemented dietary counselling on pregnancy outcome and prenatal and postnatal growth: a double-blind, placebo-controlled study (35)	RCT	Luoto R	2010	British Journal of Nutrition	Probiotic intervention reduced the risk of GDM and dietary intervention diminished the risk of larger birth size in affected cases. The results of the present study show that probiotic-supplemented perinatal dietary counselling could be a safe and cost-effective tool in addressing the metabolic epidemic.	246	4.13
Vitamin D supplementation for women during pregnancy (36)	Review	De-Regil LM	2016	Cochrane Database of Systematic Reviews	This updated review, which included two trials involving 219 women, did not find a clear difference in the risk of gestational diabetes between women receiving vitamin D supplements, those receiving no intervention, and those in the placebo group. The effects of vitamin D supplements on women with a diagnosis of gestational diabetes or with increased risk of pre-eclampsia should be assessed.	196	12.01
Low glycemic index diet in pregnancy to prevent macrosomia (ROLO study): randomized control trial (37)	RCT	Walsh JM	2012	BMJ	800 women without diabetes were randomized to either receive no dietary intervention or start on a low GI diet from early pregnancy. The low GI diet had a significant positive effect on gestational weight gain and maternal glucose intolerance.	169	38.20

### 3.5. Research hotspots and frontiers

#### 3.5.1. Keywords visualization

**Figure 4:**
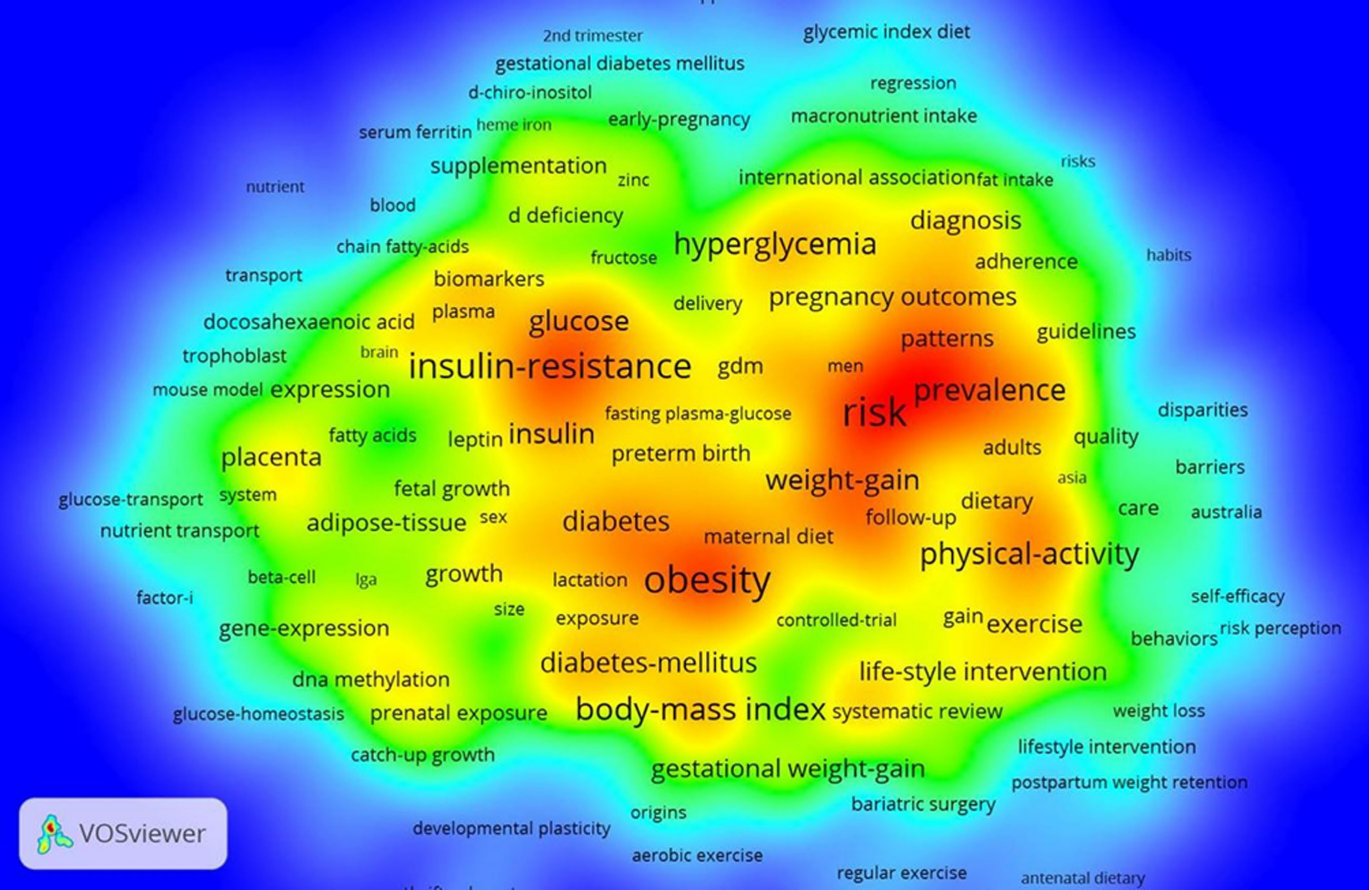
The heat map of keywords in the field of GDM and nutrition in the WoSCC database. The heat map indicates the frequency of keywords according to different shades of color; warm red represents the hot areas, and cold blue represents the cold areas.

Keywords are the core and essence of a paper. We used VOSviewer and CiteSpace to visualize the keywords, status, and future directions of this field. The keywords related to topics such as “Gestational Diabetes Mellitus,” “GDM,” “diet,” “nutrient,” and “nutrition” were excluded as they have more apropos interpretations and based on the principle that the minimum number of occurrences of keywords was five or greater, we found 609 keywords that meet the threshold. Figure 4 illustrates the high-occurrence keywords of GDM and nutrition from 2011 to 2021, and the most popular keywords were: obesity (378), risk (367), mellitus (253), insulin resistance (251), body-mass index (167), physical activity (157), prevalence (155), association (153), outcomes (143), and overweight (141).

**Figure 5:**
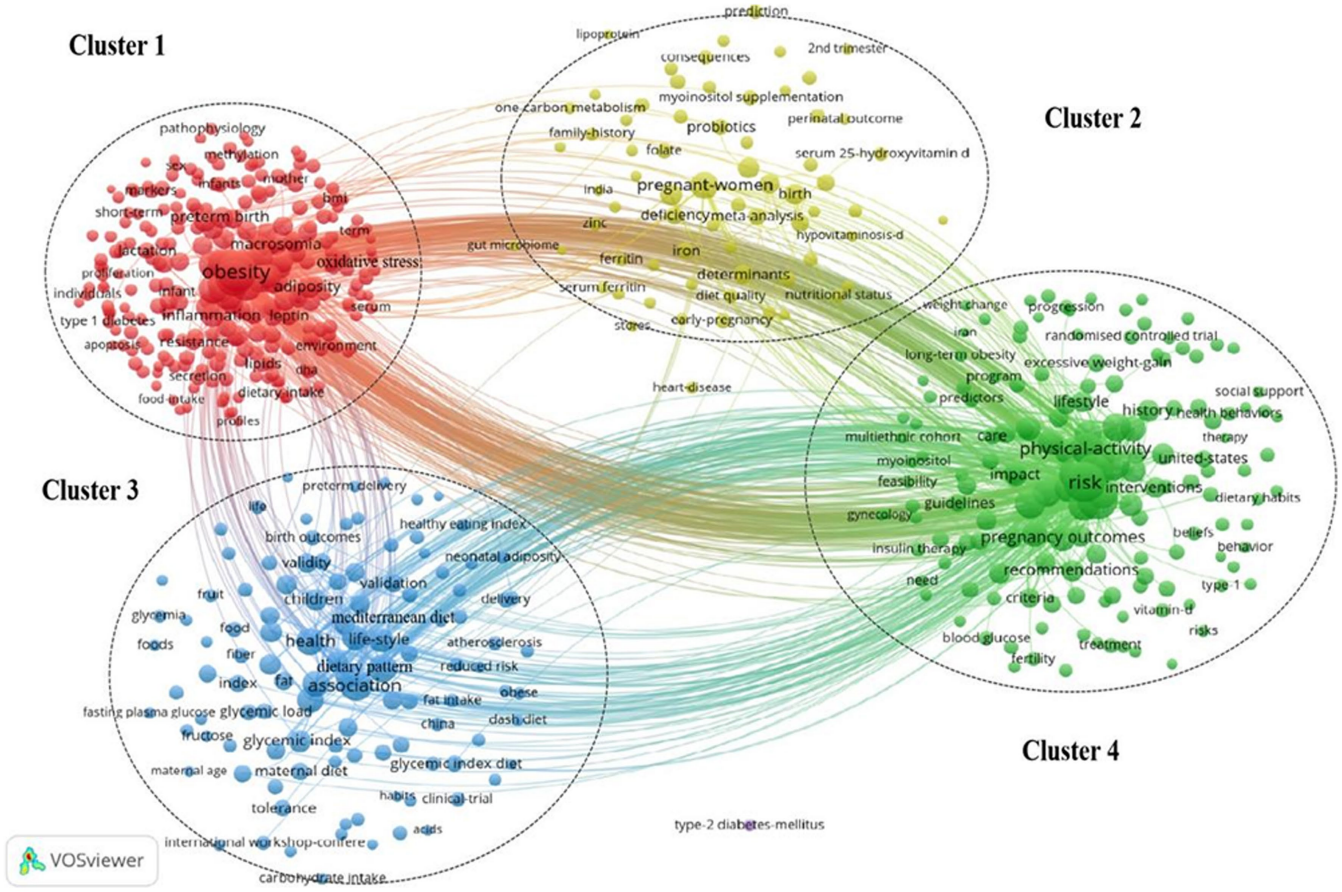
Keywords co-occurrence cluster map.

The inclusion of keywords could be categorized into four clusters based on the keywords visualization representing the current four most popular research directions in this field (Figure 5): (1) Oxidative stress inflammation biomarkers (cluster 1, 266 keywords); (2) serum 25-hydroxyvitamin D iron folate myoinositol probiotics (cluster 2, 70 keywords); (3) dietary patterns glycemic index diet Mediterranean diet meta-analysis (cluster 3, 110 keywords); and (4) risk gestational weight gain (GWG) lifestyle intervention physical activity (cluster 4 154 keywords).

#### 3.5.2. Burst keywords and new frontiers

**Figure 6:**
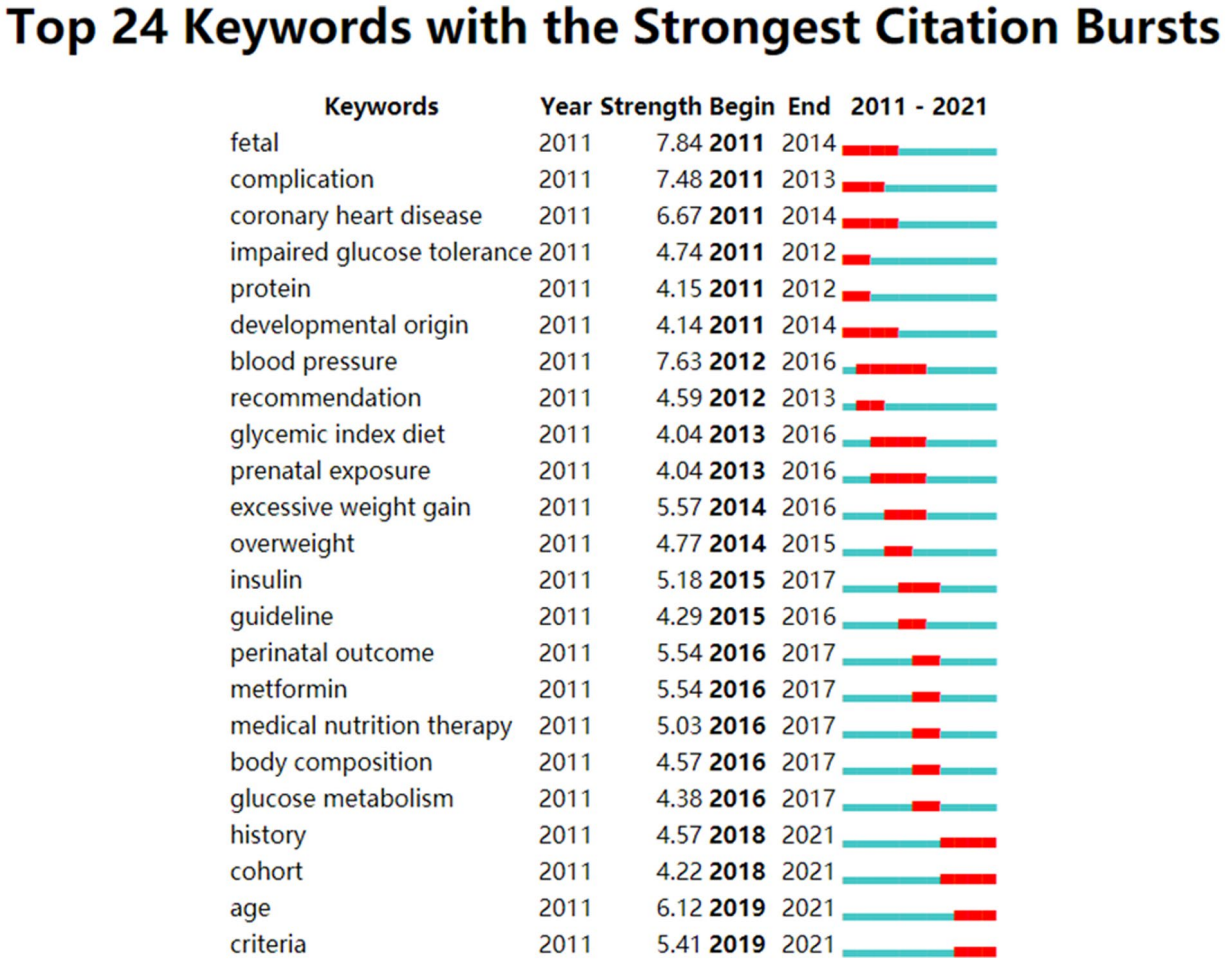
The keywords with the strongest citations bursts of publications on GDM and nutrition. The blue line represents the time interval, while the burst period is illustrated as the red segment on the blue timeline.

A “Burst” detects the literature keywords that have changed greatly in frequency within a certain period, which can last for several years or just one (38). In addition, a keywords burst analysis reveals hot points and the forward position field that often has guiding meanings (39). Figure 6 illustrates the top 24 keywords with the strongest citation bursts. The keywords that indicated the forefront of the research and lasted until 2021 were: history (2018–2021), cohort (2018–2021), age (2019–2021), and criteria (2018–2021).

## 4. Discussion

The study used visualization software to examine the research field of GDM and nutrition from 2011 to 2021. Our results suggest that GDM and nutrition is a rapidly evolving field with enormous potential and is more than the cornerstones of medical therapy. There is no doubt that this research area is a great step forward, and we are convinced that GDM and nutrition will continue to grow. The trend is expected to increase global awareness of GDM and nutrition.

### 4.1. Global potential and achievement

According to the analysis of the number of publications and citations, we found that this research field may still be a hotspot in the coming years. Overall, countries with a high frequency of publications also had high centrality, which meant the closeness of research cooperation between countries/regions. Among the top ten, five were in Europe, one was in Asia, two were in North America, and one was in Oceania. Moreover, among the top ten institutions, six were from the United States. Scholars in the United States seemed to have more achievements, whether in quantity or centrality, and they benefited from the excellent research environment and generous financial situation in this country. Surprisingly, the relatively high-frequency publications were demonstrated while lacking good research cooperation relationships with other countries, China had a centrality of only 0.04. As a developing country, China has not yet invested in costly basic scientific research, for example, in GDM molecular biology screening techniques, due to the challenge of unfavorable political factors (40), resource allocation (41), and national research priority (42). Particularly, research funding was limited, and the medical resources in developing countries are limited, including the lack of available labs and trained phlebotomists to collect vein blood samples in remote rural areas of developing countries (43). It was important to encourage collaborative efforts among countries with different economic resources to strengthen high-quality research in GDM and nutrition further.

### 4.2. Research energy and potential

In terms of high-impact authors and cooperative relationships, we found that the top three authors by frequency of publications, co-citations, and *H*-index were from the United States. Similar results were found in GDM research (44), and overall, we consider the United States as the leading country in this field. It was the most productive country and participated in the most collaborative efforts. Although there is a lack of competent evidence and data, the United States has a leading role in most fields of medicine (45). Through our research, the most productive authors, Zhang, Bao, and Tobias, had nine publications and collaborated closely. Zhang was regarded as the leader in this field, whose *H*-index reached 20, and the average citation was 47.03 per paper. He focused on the large sample, population-based cohort, and prospective studies with long research periods. Additionally, he provided relatively reliable evidence to prove the causality of diseases, for example, different dietary patterns, nutrients, and undiscovered risks among women with a history of GDM (33, 46–48). Interestingly, neither the top 10 authors with a high frequency of publications nor the co-cited authors had any scholars from China. This is likely because (1) Chinese scholars have not focused on this field, and (2) Chinese scholars’ poor language capacity resulted in low-level quality. It is imperative that Chinese scholars invest more in this area and publish more high-quality papers. It is also important that eliminate academic barriers and promote GDM and nutrition research development.

There were four studies consisting of RCT or meta-analyses of RCTs and one review featured in the top five items of cited literature. Thangaratinam et al. and Poston et al. (32) were the authors of the most cited papers, with 549 and 420 citations, respectively. Their research demonstrated the effectiveness of a mixed approach based on nutrition and diet in reducing maternal GWG and improving pregnancy outcomes in pregnant and obese women. Both studies emphasized the importance of nutritional interventions in the field of GDM for specific populations (32, 34). Luoto et al. (35) conducted an RCT study of 256 women and indicated probiotic intervention to reduce the risk of GDM, highlighting the potential of probiotic-supplemented perinatal dietary counseling as a safe and cost-effective tool in addressing the metabolic epidemic during pregnancy. De-Regil et al. (36) reviewed two trials involving 219 women and did not find a significant difference in the risk of GDM between women receiving vitamin D supplements, those receiving no intervention, and the placebo group. Walsh et al. (37) designed an RCT study with 800 women and demonstrated that a low GI diet had a significant positive impact on gestational weight gain and maternal glucose intolerance.

### 4.3. Research hotspots and frontiers

We visualized keyword occurrence frequency and time trends using VOSviewer and CiteSpace (24–28). Based on a cluster analysis and the strongest citation bursts of the keywords, the current research hotspots were mainly categorized into four clusters.

#### 4.3.1. The effects of different nutrients on biomarkers and pregnancy outcomes in GDM

In this study, an important research hotspot and direction was pathophysiology in GDM (Figure 6, cluster 1). Insulin Resistance (IR) increases in the late stage of the second trimester to levels near that regarded as Type 2 Diabetes Mellitus (T2DM) in normal pregnancy (49, 50). Most pregnant women maintain normoglycemia glycosuria due to higher insulin secretion adequate for *β*-cell compensation (49). However, GDM occurs if IR for *β*-cell compensation is inadequate (49, 51), and it may be featured with other abnormalities in adipokine and cytokine dysfunction. Adipokine is seen as one of the biomarkers and provides potential links between obesity and IR (49) in pregnancy with GDM, and they also have the characteristic of chronic, low-level inflammation (52). In addition, oxidative stress is the normal factor, which hides IR (53). Oxidative stress induces many pathways which generate inflammation; additionally, the various pathways that lead to released inflammatory mediators (e.g., adhesion molecules and interleukins) are all induced by oxidative stress (54, 55).

There was much evidence suggesting that different nutrients play a role in inflammation, oxidative stress, and other biomarkers, resulting in pregnancy outcomes in GDM, such as improving insulin sensitivity and limited pathways of lipid profiles. Thiamin, which is known as vitamin B1, is a nutrient necessary for the complicated steps of anti-inflammatory action and lipid and glucose metabolism, which then facilitates glycemic control of gestational diabetes (56, 57). In a study conducted by Amirani et al., thiamin supplementation significantly reduced C-reactive protein (CRP) and malondialdehyde (MDA) levels and gene expression of TNF-α to pregnancy with GDM for 6 weeks (58). Recent studies have demonstrated that Vitamin E (58) and omega-3 fatty acid (31) levels were lower in GDM women than in healthy pregnant women. A study by Jamilian et al. observed that vitamin E and omega-3 fatty acid co-supplementation, compared with the placebo group, resulted in a significant increase in total antioxidant capacity levels and nitric oxide and a significant decrease in plasma MDA concentrations and then lower incidences of hyperbilirubinemia in newborns (59). GDM has a higher risk of micronutrient insufficiency than normoglycemia individuals (60, 61). A randomized, double-blinded, placebo-controlled trial was conducted on 60 pregnant women with GDM and indicated that magnesium-zinc-calcium co-supplementation for 6 weeks might decrease serum high-sensitivity CRP, total plasma nitrite, and MDA levels (62). Some studies have reported that body selenium status plays a key role in glucose homeostasis (63, 64), anti-inflammation (65), and oxidative stress (66, 67) in patients with GDM. A study from Asemi et al. demonstrated that GDM women who took selenium supplements were associated with reduced high-sensitivity CRP levels in serum; however, surprisingly, this did not influence nitric oxide concentrations in plasma (68).

#### 4.3.2. Deficits or excesses of micronutrients contribute to the development of GDM

In the past decade, various nutritional intervention strategies have been used to reduce the risk of GDM. Generally, reasonable dietary micronutrients and supplements can provide a feasible option for preventing and treating GDM. In this study, the role of some micronutrients, such as vitamin D, myoinositol, iron, folate, and probiotics for the risk of GDM, was formed as one of the hotspots (Figure 6, cluster 2).

Insufficient supply of Vitamin D is common among pregnant women and has been a greater risk for some pregnancy complications such as GDM (69, 70). A meta-review of 20 studies, including 16,515 patients, researching the effect of vitamin D deficiency manifested that it could raise GDM risk by 45% (71). Zhang et al. noted that vitamin D deficiency increased the plasma glucose level among pregnant women who were overweight and obese, which also increased the risk of GDM (72). Myoinositol is an isomer that influences the body’s insulin response and several hormones associated with T2DM (73). Dietary intake should ideally contain 1 g/day of myoinositol from grain, meat, fresh fruits, vegetables, corn, and legumes (74). A secondary analysis based on three randomized, controlled trials (595 patients who were at risk as GDM, obese, and overweight) provided myoinositol (4 g/d) throughout pregnancy, and the results demonstrated that the ratio of GDM and the risk of premature birth and macrosomia in the women with risk factors of GDM were reduced (75). Adequate iron is crucial for the function of β-cell and glucose homeostasis, but excess endogenous or exogenous (supplemental) iron is associated with GDM remains controversial (76). Most recently, Zhang et al. published a systematic qualitative review on dietary iron intake and iron status and demonstrated that iron intake, particularly heme-iron, was significantly and positively associated with GDM during or before pregnancy, even adjusting the confounder as primary dietary factors and other well-documented risk factors of GDM (77). Another quantitative meta-analysis of the relationship between dietary iron intake, iron supplementation, and circulating iron biomarkers with GDM found neither dietary iron (non-heme iron) nor supplemental iron intake was associated with an increased odd ratio for GDM (78). Folate (vitamin B9) and vitamin B12 play a role in metabolism of one-carbon, which is associated with the disruption of DNA synthesis, cellular inflammation, and adiposity dysfunction, which might lead to glucose intolerance (79). However, studies had suggested a relationship between folate and vitamin B12 status and GDM, particularly if there was an imbalance between folate and vitamin B12, with high folate and low vitamin B12 (80, 81).

The diversity of the gut microbial population and its essential role in inflammation, adiposity, and glucose intolerance in women with GDM (82–84). Microbiota-targeted strategies, such as probiotics, are defined as “live microorganisms which, when administered in inadequate amounts, confer a health benefit on the host” by the World Health Organization and could enhance healthy outcomes in GDM (85). A meta-analysis by Chen et al. included seven studies and indicated that probiotics supplementation reduces fasting glucose in pregnant women with GDM (86), but not all probiotics handle similar clinical benefits (87). Hence, it could be a hotspot for future research to test the personalized and precise probiotics supplementation, considering the interaction with host gut microbiota composition and diet in GDM.

#### 4.3.3. Toward a holistic approach to dietary management of GDM

Whilst the traditional approach of examining diseases in relation to diet by focusing on single nutrients has been valuable, it is subject to a range of conceptual and methodological limitations (88). Another dietary assessment method is that of “dietary patterns,” which considers the complex interrelationships between different foods and nutrients as a whole (88, 89). Dietary patterns are “the quantities, proportions, variety or combination of different foods, drinks, and nutrients (when available) in diets and the frequency with which they are consumed (90).” They are shaped by sex, socioeconomic status (88), individual preference and beliefs, as well as geographical and environmental factors (91). Dietary patterns are not set in stone because of changes in food preferences and availability (88). Moreover, there is growing evidence that food-based analysis methods that incorporate single nutrient and dietary patterns comprehensively reflect dietary preferences but can also promote health and predict chronic disease risk more accurately (92). However, there is a paucity of studies pertaining to the diverse forms of dietary patterns in relation to GDM, which indicates that this research methodology may emerge as a nascent research frontier in the future (Figure 6, cluster 3). A low glycemic index (GI) diet intrinsically promotes reasonable macronutrient intake and a high-nutrient-density nutrition. The first randomized controlled trial referred to the effectiveness of a low GI diet for GDM (*n* = 63) and found that for subjects in the low GI group the need to start using insulin reduced by 50% (93). In GDM, a low-carbohydrate diet has been proven to reduce the risk of postprandial hyperglycemia, fetal glucose exposure, and fetal overgrowth (94, 95). However, a well-matched, randomized, pilot clinical trial conducted by Hernandez et al. demonstrated the potential for improving glycemic levels and metabolic parameters with a high carbohydrate diet with more complex but low GI carbohydrates as opposed to a simple carbohydrate diet. This result clearly indicates the significance of the type and quality of the carbohydrate (96). Results from trials with caloric restriction (CR) concerns for patients with GDM have been limited. Two previous randomized trials implemented a moderate CR diet in overweight or obese pregnant women with GDM lowered glycemia levels without inducing maternal ketosis or restricting fetal growth (97, 98). The Mediterranean Diet (MD) is a pattern of eating that emphasizes plant-based foods and healthy fats (99). A study conducted on 874 early pregnant women (at 8–12 weeks in gestation) who had adopted an MD in Spain found that the MD reduced the incidence of GDM and some perinatal outcomes, including preterm birth, emergency cesarean sections, perineal trauma, and large gestational age (100).

In summary, investigating dietary patterns could have important public and clinical implications (101), since the practice of recommending foods or food groups that promote health may be more feasible than concentrating on numerous individual nutrients (102). Another strength of this analysis is the assessment of an adherence to specific dietary patterns (103). This analysis can also enhance our understanding of the complex relationship between human diets and health, and provide guidance for interventions, treatments, and education (88). For this reason, the development and promotion of healthy dietary patterns suitable for GDM will require concerted efforts from scientists, clinicians and public policy makers.

#### 4.3.4. Prevalence, risk factors, and therapeutic strategies of GDM

The prevalence, risk factors, and intervention strategies of GDM formed the fourth hotpot in this study (Figure 6, cluster 4). Overall, the prevalence of GDM was highest in the Middle East and North Africa, with a median of 15.2% (inter-quartile range 8.8–20.0%), followed by Southeast Asia, the Western Pacific, South and Central America, Sub-Saharan Africa, North America, and the Caribbean (median prevalence 15.0, 10.3, 11.2, 10.8, 7.0%, respectively), and Europe had the lowest prevalence (median 6.1%; range 1.8–31.0%) (16, 104). Although the current prevalence of GDM varies considerably, it is difficult to compare the prevalence across counties and regions worldwide due to a lack of available diagnostic testing and uniform diagnostic criteria for GDM (16).

Traditionally, some risk factors for GDM, such as advanced maternal age (105), previous history of GDM and fetal macrosomia, family history of T2DM (106), Polycystic Ovarian Syndrome (PCOS) (107), hypothyroidism (108), pre-pregnancy overweight and obesity, and genetic factors (109) are well known (15). As mentioned above, variability in the prevalence of GDM had been reported among different countries/ethnicities, even when the same diagnostic criteria were applied (16), which may be due to variations in the geographical distribution (110). Moreover, notable ethnic differences were also observed in the prevalence of GDM (111). Excess GWG is seen as a risk factor for GDM, which is major and modifiable. Excess GWG, regardless of pre-pregnancy BMI, is defined as the amount of weight gained during conception and before the infant’s birth (112), which results in a high risk of future T2DM and cardiovascular disease in GDM patients and complicates the dietary management (113). Environmental and psychosocial risk factors may play a role in developing GDM. For example, long-term exposure to persistent organic pollutants and perfluorooctanoic acid has been associated with an increased risk of GDM (114, 115). In addition, depression during early pregnancy has been prospectively associated with higher GDM risk in the future (116).

The lifestyle factors in the whole pregnancy, including eating patterns, physical activity, and glycemic control indicated by several population-based studies, appear to be important in the prevention and therapeutic strategies of GDM (117). Although it was widely accepted that medical nutrition therapy was the footstone for managing GDM, there is limited evidence on the availability of specific nutritional strategies such as total energy intake or nutrient profile (118, 119). Developing different nutrient management strategies for different types of GDM pregnant women were supposed to be one of the hotspots, just as individualized dietary management for overweight and obesity among GDM women (120, 121). The goals of optimized dietary advice for GDM include adequate intake of nutrients to ensure normal fetal growth and maternal health, but weight gain and glycemic control should be within a reasonable range during pregnancy (122). Usually, physical activities are combined with other lifestyle interventions, such as diet and drugs, rather than used separately as in the designed research protocols, so it is difficult to identify the individual contributions from physical activities (123). Generally, the effect of physical activity may largely depend on reducing excess GWG (124). There was no mutual recognition on the types, duration, and frequencies of physical activity that would be beneficial or even optimal for GDM (123). Further, larger, well-designed, and population-based cohort trials are necessary to assess the intervention strategies for improving health outcomes of women with GDM and their offspring in the short and long term.

#### 4.3.5. Currently, forming the unified diagnosis standard of GDM is the frontier of research

It is difficult to quantify and compare the reported epidemiological of GDM and draw meaningful conclusions since the controversies of diagnostic criteria continue globally, including many fundamental questions, such as when and how to conduct GDM screening using a one-step procedure or two-step procedure (125). Based on the available large-scale epidemiological data (the “HAPO study”) (126) and randomized controlled trials that have referred to the hyperglycemia diagnostic thresholds related to pregnancy complications, the International Association of Diabetes and Pregnancy Study Groups (IADPSG) criteria in 2010 was the most widely admired (2). However, the IADPSG criteria are not suitable for uniform worldwide application because of several factors, such as the availability of infrastructure, cost considerations, and dissemination of information in low- and middle-income countries (43). The Federation of Gynecology and Obstetrics (FIGO) in 2015 recommends a more flexible way to allow for dividing diagnostic processes and glucose thresholds in specific geographic regions and racial groups (113, 127). The long-term impact of the GDM label is increasingly becoming part of further research, and therefore, it is likely that research in this field will remain a hotpot in the next few years.

## 5. Strengths and limitations

To our knowledge, this study was the first bibliometric analysis of the knowledge domain and research trends on GDM and nutrition in the last decade, and to some extent, we identified the future research trends, hotspots, and frontiers in this research field. However, our study also has some limitations, which should be considered when interpreting our results. First, only SCI-E and SSCI of the WoSCC database were included in this study, which might result in ignoring other high-quality literature in the databases in this field. Second, we only introduced English publications into our analysis due to the authors’ deficient language skills; however, we included as many important and classic publications as possible in our analysis. Lastly, some undetected bias in the selection of publications should not be ruled out; although we carefully proofread the process, some mistakes were inevitable.

## 6. Conclusion

In conclusion, we discussed the research progress, hotspots, and frontiers of the GDM and nutrition field in the past decade based on information visualization technology. It was important to strengthen the collaboration between nations with different economies to produce more high-quality research on GDM and nutrition. The current research hotspots were mainly categorized into four clusters and formed four hotspots in this field: The effects of different nutrients on biomarkers and pregnancy outcomes in GDM; how deficits or excesses of micronutrients contribute to the development of GDM; toward a holistic approach to dietary management of GDM; and the prevalence, risk factors, and therapeutic strategies of GDM. The results would be helpful for professional researchers to understand the recognition modes and trends visually. Forming a unified diagnosis standard of GDM as new research perspectives for GDM and nutrition may benefit etiological research and the diagnosis and treatment of GDM.

## Data availability statement

The original contributions presented in the study are included in the article/supplementary files, further inquiries can be directed to the corresponding author.

## Author contributions

LF and YR contributed to formulating the overarching research goals and aims of this study. ZH, QC, and ML developed the method. ZH and JX analyzed and visualized the data and wrote the initial draft of the manuscript. LF and YR revised the synthesis. All authors read and approved the final manuscript.

## Funding

This work was supported by the General Research Program of Medical and Hygiene from the Health and Family Planning Commission in Zhejiang Province (2021KY746).

## Conflict of interest

The authors declare that the research was conducted in the absence of any commercial or financial relationships that could be construed as a potential conflict of interest.

## Publisher’s note

All claims expressed in this article are solely those of the authors and do not necessarily represent those of their affiliated organizations, or those of the publisher, the editors and the reviewers. Any product that may be evaluated in this article, or claim that may be made by its manufacturer, is not guaranteed or endorsed by the publisher.
